# Changes in Isoflavone Profile from Soybean Seeds during *Cheonggukjang* Fermentation Based on High-Resolution UPLC-DAD-QToF/MS: New Succinylated and Phosphorylated Conjugates

**DOI:** 10.3390/molecules27134120

**Published:** 2022-06-27

**Authors:** Suji Lee, Ryeong Ha Kwon, Ju Hyung Kim, Hyemin Na, So-Jeong Lee, Yu-Mi Choi, Hyemyeong Yoon, So Young Kim, Yong-Suk Kim, Sang Hoon Lee, Seon Mi Yoo, Heon-Woong Kim, Chi-Do Wee

**Affiliations:** 1 Department of Agro-Food Resources, National Institute of Agricultural Sciences, Rural Development Administration, Wanju 55365, Korea; sujiseven@naver.com (S.L.); haha9733@naver.com (R.H.K.); ddong128@naver.com (J.H.K.); na970713@naver.com (H.N.); quizhalo@naver.com (S.-J.L.); foodksy@korea.kr (S.Y.K.); sppringan@korea.kr (S.H.L.); yoosm@korea.kr (S.M.Y.); 2 Department of Food Science and Technology, Jeonbuk National University, Jeonju 54896, Korea; kimys08@jbnu.ac.kr; 3 National Agrobiodiversity Center, National Institute of Agricultural Sciences, Rural Development Administration, Jeonju 54874, Korea; ymchoi@korea.kr (Y.-M.C.); hyemyeung1@korea.kr (H.Y.)

**Keywords:** soybean seed, fermentation, *cheonggukjang*, isoflavone, succinyl-glucosides, phosphorylated conjugates, UPLC-DAD-QToF/MS

## Abstract

In this study, thirty-eight isoflavone derivatives were comprehensively identified and quantified from the raw, steamed and fermented seeds of four selected soybean cultivars based on UPLC-DAD-QToF/MS results with reference to the previously reported LC-MS library and flavonoid database, and summarized by acylated group including glucosides (Glu), malonyl-glucosides (Mal-Glu), acetyl-glucosides (Ac-Glu), succinyl-glucosides (Suc-Glu) and phosphorylated conjugates (Phos) in addition to aglycones. Among them, Suc-Glu and Phos derivatives were newly generated due to fermentation by *B. subtilis* AFY-2 (*cheonggukjang*). In particular, Phos were characterized for the first time in fermented soy products using *Bacillus* species. From a proposed roadmap on isoflavone-based biotransformation, predominant Mal-Glu (77.5–84.2%, raw) decreased rapidly by decarboxylation and deesterification into Ac-Glu and Glu (3.5–8.1% and 50.0–72.2%) during steaming, respectively. As fermentation continued, the increased Glu were mainly succinylated and phosphorylated as well as gradually hydrolyzed into their corresponding aglycones. Thus, Suc-Glu and Phos (17.3–22.4% and 1.5–5.4%, 36 h) determined depending on cultivar type and incubation time, and can be considered as important biomarkers generated during *cheonggukjang* fermentation. Additionally, the changes of isoflavone profile can be used as a fundamental report in applied microbial science as well as bioavailability research from fermented soy foods.

## 1. Introduction

Isoflavones are mainly distributed as *O*-glycosyl or *C*-glycosyl derivatives with acylated moieties (e.g., none-, acetyl- and malonyl-) in soybean, kudzu, astragalus and red clover belonging to Leguminosae Family [1,2] and have been reported to have potential preventive effects in a wide range of human diseases such as cancer, hypertension, atherosclerosis, diabetes, hyperlipidemia, menopausal symptoms, osteoporosis, neurodegeneration and multiple sclerosis [1,3,4]. Particularly, soybean (*Glycine max* L.), commonly known as an isoflavone-rich source, is one of the most important crops consumed worldwide because it provides the functional nutrients (vegetable protein, oil, fatty acid, isoflavone, saponin, etc.) and related health benefits through dietary soy-based foods (e.g., bean sprouts, soymilk, soy paste, tofu, yuba, *miso*, *natto*, *doenjang* and *cheonggukjang*) [5,6,7]. In general, *cheonggukjang* and *natto*, which are representatively traditional fermented foods in Korea and Japan, respectively, are produced under different *Bacillus* species (*B. subtilis*, *cheonggukjang*; *B. subtilis* natto, *natto*) and processed conditions during fermentation [8,9].

According to previous reports, malonyl-glucosides predominantly found in raw soybeans can be decarboxylated and deesterified into acetyl-glucosides and glucosides, respectively, by thermal procedures including steaming, baking and roasting [10,11]. Additionally, as fermentation continued, the increased glucosides after steaming were hydrolyzed to the corresponding aglycones by certain *Bacillus* species with high β-glucosidase activity, and followed by biosynthesized into new derivatives of succinyl-glucosides and phosphorylated conjugates [12,13,14]. Considering these microbial biotransformations, most isoflavone studies during *cheonggukjang* and *natto* fermentations have been focused on the changes in their profiles under affected by type and incubation time of microorganisms as well as cooking temperature [13,15,16,17].

It has been reported that the malonyl-glucosides decreased, while the glucosides increased depending on heat-treated temperature and time during steaming and roasting, which is required in soybean cooking [7,11,18,19]. Moreover, when *cheonggukjang* and *natto* were fermented, the enzymatically 6″-*O*-acylated glycosides were newly identified as 6″-*O*-succinyldaidzin, 6″-*O*-succinylgenistin and 6″-*O*-succinylglycitin, accounting for 4.8, 7.2 and 0.6% of the total isoflavones (**TIF**s, *natto*), respectively, based on mass (MS) and nuclear magnetic resonance (NMR) spectroscopies, and found to be derivatives to play a preventive role on bone loss of an ovarian hormone-deficiency rat model [8,9]. Among them, 6″-*O*-succinyldaidzin of Japanese *natto* elicited anti-ischemic effects by reducing both infarct areas and neurological scores of the middle cerebral artery occlusion rats [20]. The Korean *cheonggukjang* presented significantly clinical effects in hyperlipidemia-induced hamsters [21], and 6″-*O*-succinyldaidzin and 6″-*O*-succinylgenistin derived from these foods were rapidly accumulated after 15–18 h prior to maximum conversion after 27–36 h during their fermentation [22,23].

Recently, Kwon et al. [24] identified and quantified a total of eight succinylated derivatives (20.3% and 13.6% of **TIF**s), including additional 4″-*O*-succinyldaidzin and 4″-*O*-succinylgenistin from commercially available *cheonggukjang* and *natto* using high-resolution QToF-MS coupled with UPLC-DAD system. As phosphorylated isoflavones, four conjugates were newly generated via biotransformation on the growth of *B. subtilis* and *B. amyloliquefaciens* cultivated with pure standards (daidzein, genistein, daidzin and genistin), and determined as 7-*O*-phosphate or 4′-*O*-phosphate based on daidzein and genistein by MS fragmentation and NMR elucidation [14,25]. However, these conjugates have not been directly characterized from the fermented soy foods. Other isomers associated with 6″-*O*-succinyl-glucosides are still limited in their detailed changes with different fermented conditions. Since the simplified isoflavone structures caused by fermentation lead to faster absorption and higher bioavailability after consumption of *cheonggukjang* in human clinical studies [26], it is also necessary to provide a comprehensive profile in the changed isoflavone derivatives.

Thus, in this study, a comprehensive isoflavones profiling was precisely performed through spectral interpretation of QToF-MS with reference to previously reported LC-MS library and flavonoid database (RDA DB 1.0 published in 2016) from the raw, steamed and fermented soybean seeds. Additionally, it was purposed to evaluate the isoflavone changes including primarily succinylated and phosphorylated conjugates during steaming and *cheonggukjang* fermentation (by *B. subtilis* AFY-2) with four selected cultivars based on UPLC-DAD. In order to regulate key metabolites during steaming and fermentation, a roadmap on biotransformation of soy isoflavones could also be proposed consisting of these completed profiles. Finally, the profile of isoflavone changes provided in the present study suggest that the presence of succinylated and phosphorylated isoflavones can be considered as important biomarkers during fermentation with *Bacillus* species, and can be used as a fundamental report in advanced microbial science as well as human bioavailability research after consumption of fermented soy foods.

## 2. Results and Discussion

### 2.1. Identification of 38 Isoflavone Derivatives in Raw, Steamed and Fermented Soybean Seeds

**Figure 1:**
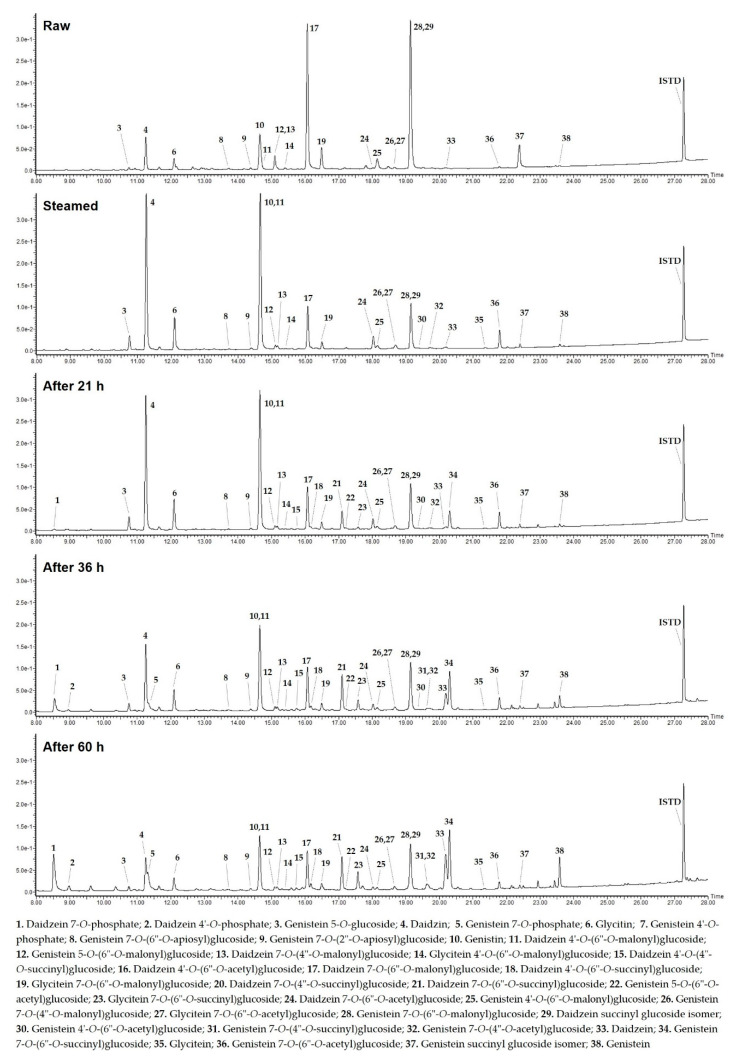
UPLD-DAD chromatograms of 38 isoflavone derivatives (wavelength at 254 nm) from the raw, steamed and fermented (after 21, 36 and 60 h) seed samples of soybean cultivar, ‘KLS 87248’.

As shown in Figure 1 and Table 1, a total of thirty-eight isoflavone derivatives were tentatively identified from the raw, steamed and fermented seed samples of four selected soybean cultivars (KLS 87248, Nongrim 51, GNU-2007-14613 and Daewon) by comparing elution order, UV spectra and MS fragments pattern provided from the present UPLC-DAD and QToF-MS spectral interpretation as well as previously reported LC-MS library and NMR-elucidated results, and mainly composed of glucosides (four), malonyl-glucosides (nine), acetyl-glucosides (seven), succinyl-glucosides (nine) and phosphorylated conjugates (four) based on daidzein, genistein and glycitein aglycones. These derivatives with good separation on UPLC-DAD chromatograms (KLS 87248, wavelength at 254 nm) (Figure 1) were fragmented in detail through positive ionization (+ESI, *m/z* [M + H]^+^) of high-resolution QToF-MS (Table 1).

Through recent high-resolution MS technologies, the positive ionization makes it easier to distinguish the parent molecules than conventional negative ionization due to adductive sodium (Na^+^, 23 Da) and potassium (K^+^, 39 Da) ions with hydrogen (H^+^, 1 Da), when some mixed flavonoid and phenolic acid derivatives are complexly fragmented at low concentrations [24,27,28].

#### 2.1.1. Glucosides, Malonyl-Glucosides and Acetyl-Glucosides in Raw and Steamed Seeds

From the raw and steamed soybean seeds, twenty-two glycosides were detected as structures in which glucosides (**Glu**, 162 Da; peaks **3**, **4**, **6** and **10**), malonyl-glucosides (**Mal-Glu**, 248 Da; peaks **11**–**15**, **17**, **19**, **26** and **28**), acetyl-glucosides (**Ac-Glu**, 204 Da; peaks **16**, **22**, **24**, **27**, **30**, **32** and **36**) and apiosyl-glucosides (**Api-Glu**, 294 Da; peaks **8** and **9**) combined to the 7-OH or 4′-OH of daidzein (**D**, *m/z* 255; peak **33**), genistein (**Gn**, *m/z* 271; peak **38**) and glycitein (**Gy**, *m/z* 285; peak **35**) (Figure 1 and Table 1).

Although peaks **17**, **19** and **28** (*m/z* 503, 533, 519 [M + H]^+^, based on **D**, **Gy** and **Gn**, respectively) have been typically assigned as predominant components corresponding to ‘7-*O*-(6″-*O*-malonyl)glucoside’ from soybean seeds, the unknown isomers closely related to these compounds were still traced in ESI-MS selected ion chromatogram under affected by varietal differences [29]. Two malonylated isomers were further confirmed as ‘daidzein 7-*O*-(4″-*O*-malonyl)glucoside’ (peak **13**, 4″-*O*-malonyldaidzin) and ‘genistein 7-*O*-(4″-*O*-malonyl)glucoside’ (peak **26**, 4″-*O*-malonylgenistin) specified from our previous studies [24,26,27], and also consistent with LC-MS and NMR elucidation by Yerramsetty et al. [30] following novel compounds (4″-*O*-malonylated) derived from thermal treated standard solutions (6″-*O*-malonyldaidzin and 6″-*O*-malonylgenistin). Likewise, another isomers malonylated with the 6″-OH position of 4′-*O*-glucoside were indicated by their specific retention behavior and fragmentation pattern in UPLC-QToF-MS system [31], and found to be the ‘daidzein 4′-*O*-(6″-*O*-malonyl)glucoside’ (peak **11**, 6″-*O*-malonylisodaidzin), ‘glycitein 4′-*O*-(6″-*O*-malonyl)glucoside’ (peak **14**) and ‘genistein 4′-*O*-(6″-*O*-malonyl)glucoside’ (peak **25**, 6″-*O*-malonylsophoricoside) as minor compounds [24,26,27]. Thus, six additional **Mal-Glu** isomers provided similar fragmentation of [M + K]^+^, [M + Na]^+^, [M + H]^+^ and [M + H – Mal − Glu]^+^ with major group (peaks **17**, **19** and **28**).

After steaming, **Ac-Glu** slightly contained in raw soybeans increased potentially as numerous isomers due to decarboxylation of **Mal-Glu** [32]. Seven **Ac-Glu** isomers (*m/z* 459, 489 and 475 [M + H]^+^, based on **D**, **Gy** and **Gn**, respectively) could be tentatively characterized as ‘7-*O*-(4″-*O*-acetyl)glucoside’ (peak **32**), 4′-*O*-(6″-*O*-acetyl)glucoside’ (peaks **16** and **30**) and ‘5-*O*-(6″-*O*-acetyl)glucoside’ (peak **22**) including the well-known ‘7-*O*-(6″-*O*-acetyl)glucoside’ (peaks **24**, **27** and **36**) derived from corresponding **Mal-Glu** precursor by our recent findings [24,27].

#### 2.1.2. New Succinyl-Glucosides and Phosphorylated Conjugates in Fermented Seeds

**Figure 2:**
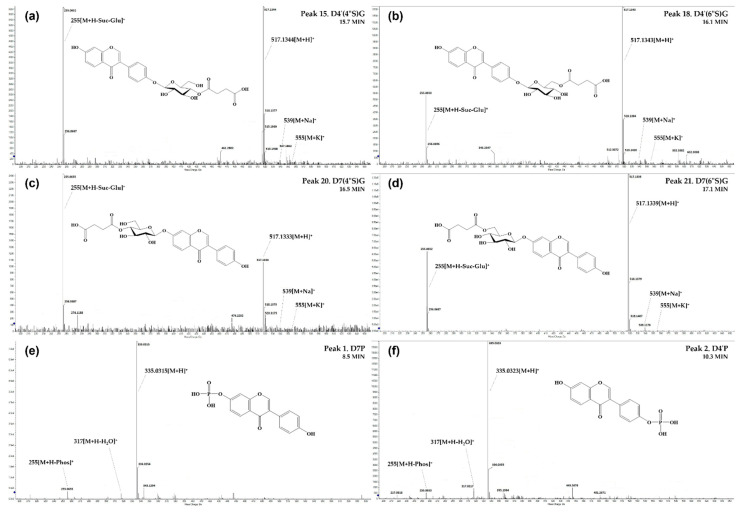
Structures and ESI(+)-QToF/MS characteristics of new succinyl-glucosides (*m*/*z* 517 [M + H]^+^) and phosphorylated conjugates (*m*/*z* 335 [M + H]^+^) based on daidzein (*m*/*z* 255 [M + H]^+^) generated from soybean seeds fermented (after 60 h) with *B. subtilis* AFY-2. (**a**) **D4**′**(4″S)G**, daidzein 4′-*O*-(4″-*O*-succinyl)glucoside; (**b**) **D4**′**(6″S)G**, daidzein 4′-*O*-(6″-*O*-succinyl)glucoside; (**c**) **D7(4″S)G**, daidzein 7-*O*-(4″-*O*-succinyl)glucoside; (**d**) **D7(6″S)G**, daidzein 7-*O*-(6″-*O*-succinyl)glucoside; (**e**) **D7P**, daidzein 7-*O*-phosphate; (**f**) **D4**′**P**, daidzein 4′-*O*-phosphate.

During *cheonggukjang* fermentation by *B. subtilis* AFY-2, succinylated and phosphorylated derivatives were newly generated from the steamed seeds [12,13], and detailed as succinyl-glucosides (**Suc-Glu**, 262 Da; peaks **15**, **18**, **20**, **21**, **23**, **29**, **31**, **34** and **37**) and phosphorylated conjugates (**Phos**, 80 Da; peaks **1**, **2**, **5** and **7**) with combination to the 7-OH or 4′-OH position of their aglycones. Overall, **Suc-Glu** studies have been primarily conducted on 6″-*O*-succinyldaidzin and 6″-*O*-succinylgenistin associated with ‘7-*O*-(6″-*O*-succinyl) glucoside’ [7,8,9,13,16,26]. However, six additional isomers, including recently reported 4″-*O*-succinyldaidzin (peak **20**) and 4″-*O*-succinylgenistin (peak **31**) [24], were fragmented from their parent ions (*m/z* 517 and 533 [M + H]^+^, based on **D** and **Gn**) to [M + H – Suc − Glu]^+^ at a low level in fermented seed samples (Figure 2a–d). In particular, daidzein-succinylated 4′-*O*-glucoside isomers as presented in Figure 2a,b had similar characteristics with the elution order (5.60 min = 4″-*O*-malonylated > 5.88 min = 6″-*O*-malonylated) and fragment ion’s relative abundance (aglycone = [M + H-Mal-Glu]^+^ > parent = [M + H]^+^, in 4″-*O*-malonylated) that Yerramsetty et al. [30] and Zhang et al. [31] reported before.

Accordingly, peaks **15** and **18** were tentatively identified as ‘daidzein 4′-*O*-(4″-*O*-succinyl)glucoside’ (**D4′(4″S)G**, 4″-*O*-succinylisodaidzin) and ‘4′-*O*-(6″-*O*-succinyl)glucoside’ (**D4′(6″S)G**, 6″-*O*-succinylisodaidzin), and have not been reported in this source yet. Interestingly, the presence of these isomers suggests that microbial succinylation depends on the binding position of intact **Mal-Glu** and **Ac-Glu** described in raw and steamed seeds.

New conjugates phosphorylated to aglycones were found to be structures of daidzein 7-*O*-phosphate/4′-*O*-phosphate (peaks **1**/**2**, *m/z* 335 [M + H]^+^) (Figure 2e,f) and genistein 7-*O*-phosphate/4′-*O*-phosphate (peaks **5**/**7**, *m/z* 351 [M + H]^+^) with fragment ions of [M + H-H_2_O]^+^ and [M + H − Phos]^+^. Despite complete identification of **Phos** on the growth of *Bacillus* species in addition to reference standards [14,25], thus far, there are no experimental reports on the qualitative and quantitative determination of these derivatives via microbial phosphorylation from fermented soy products.

### 2.2. Changes of 38 Isoflavone Derivatives during Steaming and Fermentation in Soybean Seeds

In the present study, the internal standard material (ISTD, 6-methoxyflavone) was used to evaluate the changes of isoflavones during steaming and fermentation (after 21, 36 and 60 h with *B. subtilis* AFY-2) in four selected soybean cultivars. The contents (mg/100 g, dry weight) of thirty-eight isoflavone derivatives are classified into acylated group (**aglycone**, **Glu**, **Mal-Glu**, **Ac-Glu**, **Suc-Glu** and **Phos**) according to their aglycones (**D**, **Gn** and **Gy**) in Table 2. **TIF**s could be distributed differently depending on cultivar type and processed condition of soybean seeds without their loss as follows: Daewon (199.0, 261.2, 255.3, 234.0 and 241.7 for raw, steamed, 21 h, 36 h and 60 h, respectively), KLS 87248 (374.5, 382.9, 378.8, 351.3 and 371.2 for raw, steamed, 21 h, 36 h and 60 h, respectively), Nongrim 51 (299.0, 305.9, 306.6, 313.3 and 318.3 for raw, steamed, 21 h, 36 h and 60 h, respectively) and GNU-2007–14613 (196.5, 184.0, 180.6, 181.2 and 191.3 for raw, steamed, 21 h, 36 h and 60 h, respectively).

#### 2.2.1. Glucosides, Malonyl-Glucosides and Acetyl-Glucosides during Steaming

**Figure 3:**
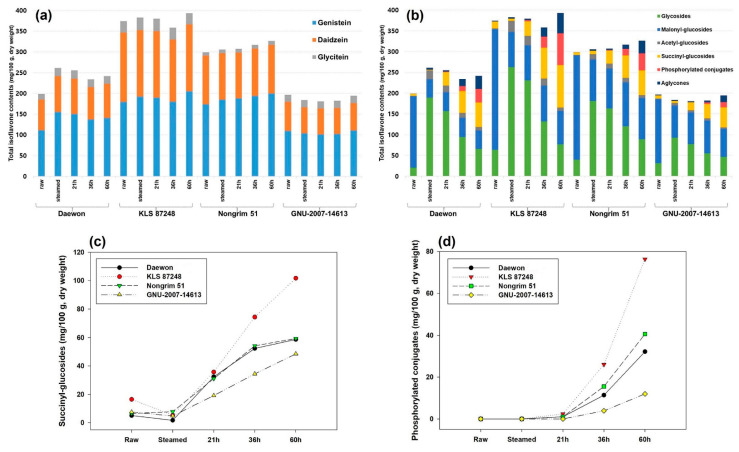
Comparison in total isoflavones content (mg/100 g, dry weight) based on (**a**) aglycone types or (**b**) derivatives as well as changes in total content of new (**c**) succinyl-glucosides and (**d**) phosphorylated conjugates from soybean samples (Daewon, KLS 87248, Nongrim 51 and GNU-2007-14613) according to steamed and fermented (after 21, 36 and 60 h with *B. subtilis* AFY-2) conditions.

Before steaming, **TIF**s of raw samples ranged from 196.5 to 374.5 agree with the seed-isoflavones variation (132.6–445.2) affected by varietal and extracted differences in previous studies [11,24,27,37]. As presented in Figure 3a,b and Table 2, the raw **TIF**s containing primarily **Mal-Glu** (77.5–84.2%) were composed of **Gn** (109.6–179.7, 54.5%), **D** (69.8–167.0, 39.0%) and **Gy** (8.0–27.8, 6.4%) based on their aglycones. The most significant change during steaming (60 min, 121 °C) is that predominant **Mal-Glu** decreased rapidly to the level of 17.0–42.1% in addition to a slight increase of **TIF**s compared to raw seeds [11,17]. In contrast, **Ac-Glu** (0–0.7%, traced) [11,27,37,38,39] and **Glu** (10.1–18.1%) were highly increased to 3.5–8.1% and 50.0–72.2% of **TIF**s, respectively, via heat-induced decarboxylation and deesterification of **Mal-Glu** [10,11,18], and are considered to play an intermediate role required for further conversion to aglycones (deglucosylation) in subsequent microbial fermentation.

#### 2.2.2. Succinyl-Glucosides and Phosphorylated Conjugates during Fermentation

As *cheonggukjang* fermentation continued up to 60 h, the increased **Ac-Glu** and **Glu** after steaming tended to decrease gradually due to their use during succinylated and phosphorylated biosynthesis [9,13,16,25] (Figure 3b). Meanwhile, it was found that the newly generated **Suc-Glu**/**Phos** not only had a close relationship with the quantitative levels of intact **TIF**s in raw cultivars, but also accounted for 9.4–12.7%/0.0–0.4% (21 h), 17.3–22.4%/1.5–5.4% (36 h) and 18.7–27.4%/4.5–14.7% (60 h) of **TIF**s according to incubated times with *B. subtilis* AFY-2. Thus, **Suc-Glu** proportions (17.3–22.4%) at 36 h of this study were similar with previous reports presented by Toda et al. [9] (12.6%, *natto*), Park et al. [13] (12.2 and 18.9%, *cheonggukjang* and *natto*) and Kwon et al. [24] (20.3 and 13.6%, *cheonggukjang* and *natto*). These results indicate that most of the isoflavone aglycones and **Glu** were converted to **Suc-Glu** within about 48 h [9] (Figure 3c). In addition, the ratio (fold) of 6″-*O*-succinylgenistin (peak **21**) to 6″-*O*-succinyldaidzin (peak **34**) as major **Suc-Glu** [8,9] were changed to 1.1–1.5 (21 h), 1.2–1.9 (36 h) and 2.0–2.7 (60 h), and implied that genistin may be succinylated faster than daidzin by *B. subtilis* AFY-2, which agrees with the results using the corresponding **Glu** standards [23].

After 36 h, **Phos** began to increase greatly with aglycones, and their content was maximized up to 60 h (Figure 3d). The detailed bioconversion rate (%) of **Phos** (mainly ‘7-*O*-phosphates’) was recently provided as a new finding; moreover, the aglycones (daidzein:genistein, 78:96) were higher than their **Glu** (daidzin:genistin, 68:90). Additionally, **Gn** could be phosphorylated more efficiently than **D** at the end of 48 h incubation with *B. subtilis* BCRC 80517 using only standard substances [25]. Through these complementary interpretations of isoflavone standards and soy products, it can be assumed that phosphorylation was delayed rather than succinylation because most of **Glu** was not converted to aglycones in the early stage (21 h) of *cheonggukjang* fermentation (Figure 3c,d). Among the cultivars used in the present study, ‘**KLS 87248**′ (Korean landrace) was succinylated and phosphorylated faster than other cultivars, and expected to be superior cultivar due to its higher **TIF**s (351.3) with **Suc-Glu** (21.1%) and **Phos** (5.4%) levels in fermentation between 36 and 48 h, which is generally required in commercially available *cheonggukjang* [8,40]. However, since **Phos** derivatives have not been detected directly from fermented soy products using *Bacillus* species, their quantification is still limited in terms of precision with high reliability.

#### 2.2.3. Proposed Roadmap on Biotransformation of Soy Isoflavones during Steaming and *Cheonggukjang* Fermentation

**Figure 4:**
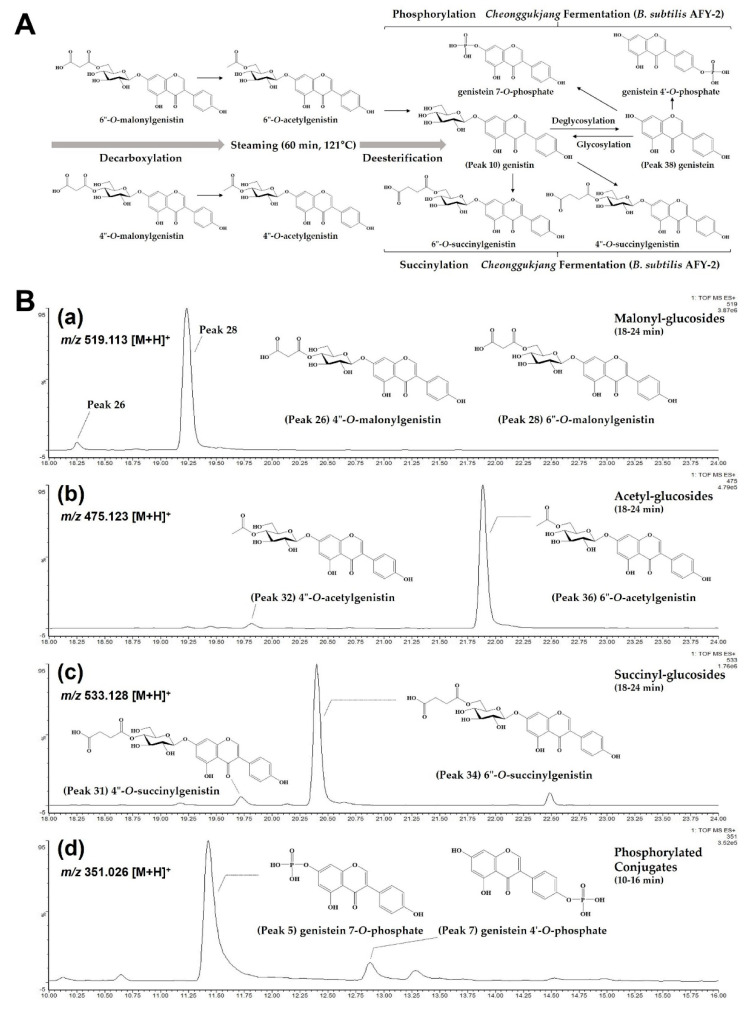
(**A**) A proposed roadmap on biotransformation of soy isoflavones during steaming and *cheonggukjang* fermentation (by *B. subtilis* AFY-2) in soybean seeds; (**B**) ESI(+)-QToF/MS (*m/z* [M + H]^+^) selected ion chromatograms of genistein-based (**a**) intact form (malonyl-glucosides, 519.113), (**b**) decarboxylation (acetyl-glucosides, 475.123), (**c**) succinylation (succinyl-glucosides, 533.128) and (**d**) phosphorylation (phosphorylated conjugates, 351.026) by bioconversion stage.

Biotransformation on soy isoflavones during steaming and fermentation (by *B. subtilis* AFY-2) could be proposed through a roadmap concluded from the changes of present 38 derivatives (**aglycone**, **Glu**, **Api-Glu**, **Mal-Glu**, **Ac-Glu**, **Suc-Glu** and **Phos**) and related previous reports [14,23,25] (Figure 4A). According to bioconversion stage (based on **Gn**), predominant **Mal-Glu** derivatives (*m/z* 519.113 [M + H]^+^) (Figure 4(Ba)) in raw seeds are decarboxylated into **Ac-Glu** derivatives (*m/z* 475.123 [M + H]^+^) (Figure 4(Bb)) and further deesterified into **Glu** during steaming. The increased **Glu** after steaming were mainly succinylated at the 6″-OH or 4″-OH positions of 7-*O*-glucoside (*m/z* 533.128 [M + H]^+^) (Figure 4(Bc)) as well as deglycosylated into aglycones during fermentation. In addition, **Phos** derivatives (*m/z* 351.026 [M + H]^+^) (Figure 4(Bd)) were newly formed by phosphorylation at the 7-OH or 4′-OH position of the converted aglycones. Therefore, isoflavone glycosides (**Mal-Glu**, **Ac-Glu** and **Glu**) were gradually hydrolyzed into their corresponding aglycones by thermal procedure (steaming) and β-glucosidase of *B. subtilis* AFY-2 (fermentation). After conversion to aglycones, it was found that the enhanced succinylation and phosphorylation varied depending on cultivar type and fermentation time. To enhance **Suc-Glu** and **Phos** additionally, different *Bacillus* species should be further characterized by means of their catalytic properties. In the future, it will be also possible to evaluate bioconversion for representative glycosidic type from other isoflavone-rich resources such as kudzu, astragalus and red clover by considering this roadmap.

## 3. Materials and Methods

### 3.1. Chemicals and Reagents

Reference standards of daidzein (≥95%), genistein (≥95%), daidzin (≥90%) and genistin (≥95%) were purchased from Sigma-Aldrich Co. (St. Louis, MO, USA); glycitein (≥98%), glycitin (≥98%), sophoricoside (≥98%) and 6-methoxyflavone (ISTD, ≥95%) were obtained from Extrasynthese (Genay Cedex, France); 6″-*O*-malonyldaidzin (≥95%), 6″-*O*-malonylgenistin (≥90%), 6″-*O*-malonylglycitin (≥95%), 6″-*O*-acetyldaidzin (≥95%) and 6″-*O*-acetylgenistin (≥95%) were obtained from Synthose Inc. (Ontario, Canada); and 6″-*O*-acetylglycitin (99%) was obtained from MedChemExpress (Monmouth Junction, NJ, USA). LC-MS grade methanol, acetonitrile and water were supplied from Thermo Fisher Scientific Inc. (Waltham, MA, USA). Additionally, LC grade formic acid (Junsei Chemical, Tokyo, Japan) was used to improve the efficiency of sample extraction and chromatographic separation.

### 3.2. Plant Materials

Among fifty-one core collected soybean cultivars with superior agronomic traits and flavonoid profiles recommended by the Gene Bank of National Agrobiodiversity Center (NAC, Jeonju, Korea), ‘**KLS 87248**′ (Korean landrace, rich seed-isoflavone), ‘**Nongrim 51**′ (Japanese breeding line, rich seed-isoflavone and leaf-flavonol), ‘**GNU-2007-14613**′ (Korean landrace, rich seed production in field) and ‘**Daewon**’ (a commercial variety commonly used in Korean *cheonggukjang*) were chosen considering their size (large) and yellow-coated color, which is required in seed types suitable for *cheonggukjang* fermentation. The selected four cultivars were sown on experimental field (19 June 2020, in rows at a spacing of 15 cm) located within the center, and cultivated under similar conditions during the country’s cropping season (June–November 2020). Their matured seeds were hand-harvested and further dried in a Bionex oven (Vision Scientific Co., Daejeon, Korea) for 72 h at 50 °C. Based on the Rural Development Administration (RDA, Jeonju, Korea) guideline, the seed size could be classified as small (<13 g), medium (13–24 g) and large (>24 g) with regard to their one hundred seeds weight [41]. Moreover, experimental research and field studies on plant materials of this study complies with relevant institutional, national and international guidelines and legislation.

### 3.3. Preparation of Cheonggukjang with B. Subtilis AFY-2

*B. subtilis* AFY-2 (KACC91988P, AFY 2) used in the present study was purchased from NUC Co. (Daegu, Korea). *Cheonggukjang* samples were prepared according to the method described by previous reports [8,16,22] with slight modification. Briefly, the washed seeds (1 kg) were soaked with 5 L of tap water for 12 h at room temperature and autoclaved for 60 min at 121 °C to conduct their steaming (Bionex VS-1221, Vision Scientific Co., Daejeon-Si, Korea). Steamed seeds were cooled down and inoculated with 1% (*w*/*w*) *B. subtilis* AFY-2, and followed by fermented for 60 h at 35 °C in an incubation room. To evaluate isoflavone changes during fermentation, *cheonggukjang* designed by incubation time was sampled at 0, 21, 36 and 60 h, respectively. The collected materials were lyophilized and finely grounded with a sample mill for use as analytical samples.

### 3.4. Extraction and UPLC-DAD-QToF/MS Analysis of Isoflavone Derivatives

With reference to Kim et al. [28] method, 1 g of powdered sample was extracted with mixed solvents (methanol:water:formic acid = 50:45:5, *v*/*v*/*v*, 10 mL) using an orbital shaker (for 30 min at 200 rpm), and continued centrifuging for 15 min at 2016× *g* and 4 °C (LABOGENE 1580R, Bio-Medical Science Co., Seoul, Korea). The collected supernatant was filtered through a 0.2 µm PVDF syringe filter (Thermo Fisher Scientific Inc., Waltham, MA, USA). Each filtrate (0.5 mL) and ISTD (6-methoxyflavone 50 ppm, 0.5 mL) were further diluted with distilled water to a final 7 mL as purpose of stable recovery during solid phase extraction (SPE). To summarize the overall SPE method, a C_18_ cartridge (Hypersep C18 500 mg, Thermo Fisher Scientific Inc., Waltham, MA, USA) was washed with methanol (3 mL), and followed by conditioned with distilled water (5 mL) for its initial activation. The previously diluted filtrate and ISTD were sequentially loaded on activated cartridge and washed with 5 mL of distilled water. Finally, the targeted compounds were slowly eluted from the loaded cartridge by 1% formic acid in methanol (5 mL). The semi-purified isoflavone eluate was completely concentrated using N_2_ gas and re-dissolved in above extraction solvent (0.5 mL) prior to UPLC-DAD-QToF/MS analysis.

A high-resolution analytical QToF/MS system (Xevo G2-S QToF, Waters MS Technologies, Manchester, UK) coupled with UPLC-DAD (ACQUITY UPLC^TM^ system, Waters Co., Miliford, MA, USA) was operated to identify and quantify isoflavone derivatives including succinyl-glucosides and phosphorylated conjugates from the raw, steamed and fermented (*cheonggukjang*) samples of soybean seeds. On the basis of our previous report [42], UPLC conditions used were set followed as: 0.5% formic acid in water (eluent A) and 0.5% formic acid in acetonitrile (eluent B) used as a mobile phase in gradient program of initial (5% B), 20 min (25% B), 25 min (50% B), 30–32 min (90% B), 35–40 min (5% B, stabilization); flow rate (0.3 mL/min); sample injection volume (1 μL); CORTECS T3 C18 column (2.1 × 150 mm, 1.6 μm, Waters Co., Milford, MA, USA) and pre-column (CORTECS UPLC T3 VanGuard™, 2.1 × 50 mm, 1.6 μm, Waters Co., Milford, MA, USA); column oven temperature (30 °C). UV spectra was multi-scanned from 210 to 400 nm (254 nm for isoflavones). Mass spectra (positive ions) were simultaneously scanned with the range of *m/z* 100–1000 through an electrospray ionization (+ESI) probe, and their key parameters were: capillary voltage 3.5 kV, sampling cone voltage 40 V, source temperature 120 °C, desolvation temperature 500 °C, desolvation N_2_ gas flow 1020 L/h. All experimental analyses were carried out in triplicates.

### 3.5. Identification and Quantification of Isoflavone Derivatives

The previously constructed LC-MS library [27] and flavonoid database (‘RDA DB 1.0-Flavonoids’ completed in 2016, Korea) [2] (pp. 99–103) were used to clearly and efficiently identify isoflavone derivatives from the soybean seed samples (raw, steamed and *cheonggukjang*-fermented), and composed of twenty compounds information including positive and negative fragment ions. The purposed isoflavones were tentatively determined by comparing the positive fragmentation, UV spectra and elution order presented in the library and database. In particular, some derivatives of them were further confirmed through consistence with 13 types of reference standards provided in Table 1. Nevertheless, since it was impossible to obtain all available standards in relation to the identified derivatives, the quantification for each compound was calculated as 1:1 without considering the relative response factor for pre-inserted ISTD (based on UV detection at 254 nm).

### 3.6. Statistical Analysis

Data were expressed as means ± standard deviations in their triplicated results. Significant differences were determined with one-way analysis of variance (ANOVA) followed by Duncan’s multiple-range test using SPSS version 27.0 software (SPSS Inc., Chicago, IL, USA). The *p*-values < 0.05 were considered statistically significant.

## 4. Conclusions

In this work, thirty-eight isoflavone derivatives were comprehensively identified and quantified from the raw, steamed and fermented seed samples of four selected soybean cultivars based on high-resolution UPLC-DAD-QToF/MS results with reference to previously reported LC-MS library and flavonoid database (RDA DB 1.0 completed in 2016), and categorized into **Glu**, **Api-Glu**, **Mal-Glu**, **Ac-Glu**, **Suc-Glu** and **Phos** as acylated group along with **D**, **Gn** and **Gy**. Especially, **Suc-Glu** and **Phos** derivatives were newly generated during *cheonggukjang* fermentation by *B. subtilis* AFY-2, and among them, **Phos** were characterized for the first time in fermented soy products using *Bacillus* species. Most of the isoflavone quantification have been accurately evaluated in variation according to variety, cropping environment, storage duration and extraction solvent system of soybeans based on only 12 types of available standards (**Glu**, **Mal-Glu**, **Ac-Glu** and aglycones) [37,39,43]. However, since **Suc-Glu** (17.3–22.4%) and **Phos** (1.5–5.4%) have a significant proportion in 36 h-fermented *cheonggukjang* products, their absence can lead to a reduction of the **TIF**s. Additionally, the role of these derivatives should be elucidated in leading to higher isoflavone bioavailability after *cheonggukjang* consumption on human clinical studies [26]. Thus, the succinylated and phosphorylated isoflavones can be considered as important biomarkers generated newly during fermentation with *Bacillus* species, and a roadmap on isoflavone-based biotransformation proposed in this study can contribute to the enhanced production of these conjugates through metabolites regulation at each bioconversion stage. In the future, it is also necessary to carry out metabolomics approach to how isoflavone derivatives change according to type and concentration of microorganisms during fermentation as well as discover potential pharmacological activities from the **Suc-Glu** and **Phos** derivatives.

## Figures and Tables

**Table 1:** Characterization of thirty-eight isoflavones from the raw, steamed and fermented (by *B. subtilis* AFY-2) soybean seeds based on UPLC-DAD-QToF/MS.

Acylated Group	Peak No.	Isoflavone Derivatives	Abbreviation	RT (min)	Molecular Formula	Observed *m/z* [M + H]^+^	Error ^(3)^ (ppm)	ESI(+)-QToF-MS (Fragmented Ions of [M + H]^+^, *m/z*)	References
***Daidzein Derivatives (14)***									
Aglycone	33 ^(2)^	Daidzein	D	20.19	C_15_H_10_O_4_	255.0652	0.0	277[M + Na]^+^, **255**[M + H]^+^	[8,19,24,26,27,29,33]
Glu	4 ^(2)^	Daidzein 7-*O*-glucoside (Daidzin)	D7G	11.26	C_21_H_20_O_9_	417.1176	−1.0	455[M + K]^+^, 439[M + Na]^+^, **417**[M + H]^+^, 255[M + H-Glu]^+^	[19,24,26,27,29,33]
Mal-Glu	11	Daidzein 4′-*O*-(6″-*O*-malonyl)glucoside (6″-*O*-Malonylisodaidzin)	D4′(6″M)G	14.65	C_24_H_22_O_12_	503.1184	0.0	541[M + K]^+^, 525[M + Na]^+^, **503**[M + H]^+^, 255[M + H-Mal-Glu]^+^	[24,26,27,31]
	13	Daidzein 7-*O*-(4″-*O*-malonyl)glucoside (4″-*O*-Malonyldaidzin)	D7(4″M)G	15.17	C_24_H_22_O_12_	503.1184	0.0	541[M + K]^+^, 525[M + Na]^+^, **503**[M + H]^+^, 255[M + H-Mal-Glu]^+^	[24,26,27,30,31]
	17 ^(2)^	Daidzein 7-*O*-(6″-*O*-malonyl)glucoside (6″-*O*-Malonyldaidzin)	D7(6″M)G	16.07	C_24_H_22_O_12_	503.1181	−0.6	541[M + K]^+^, 525[M + Na]^+^, **503**[M + H]^+^, 255[M + H-Mal-Glu]^+^	[24,26,27,29,30,31,34]
Ac-Glu	16	Daidzein 4′-*O*-(6″-*O*-acetyl)glucoside (6″-*O*-Acetylisodaidzin)	D4′(6″Ac)G	15.80	C_23_H_22_O_10_	459.1285	−0.2	497[M + K]^+^, 481[M + Na]^+^, **459**[M + H]^+^, 255[M + H-Ac-Glu]^+^	[24]
	24 ^(2)^	Daidzein 7-*O*-(6″-*O*-acetyl)glucoside (6″-*O*-Acetyldaidzin)	D7′(6″Ac)G	18.02	C_23_H_22_O_10_	459.1285	−0.2	497[M + K]^+^, 481[M + Na]^+^, **459**[M + H]^+^, 255[M + H-Ac-Glu]^+^	[8,24,26,29,35]
Suc-Glu	15 ^1)^	Daidzein 4′-*O*-(4″-*O*-succinyl)glucoside (4″-*O*-Succinylisodaidzin)	D4′(4″S)G	15.74	C_25_H_24_O_12_	517.1344	0.7	555[M + K]^+^, 539[M + Na]^+^, **517**[M + H]^+^, 255[M + H-Suc-Glu]^+^	
	18 ^(1)^	Daidzein 4′-*O*-(6″-*O*-succinyl)glucoside (6″-*O*-Succinylisodaidzin)	D4′(6″S)G	16.16	C_25_H_24_O_12_	517.1343	0.5	555[M + K]^+^, 539[M + Na]^+^, **517**[M + H]^+^, 255[M + H-Suc-Glu]^+^	
	20	Daidzein 7-*O*-(4″-*O*-succinyl)glucoside (4″-*O*-Succinyldaidzin)	D7(4″S)G	16.52	C_25_H_24_O_12_	517.1333	−1.7	555[M + K]^+^, 539[M + Na]^+^, **517**[M + H]^+^, 255[M + H-Suc-Glu]^+^	[24]
	21	Daidzein 7-*O*-(6″-*O*-succinyl)glucoside (6″-*O*-Succinyldaidzin)	D7(6″S)G	17.10	C_25_H_24_O_12_	517.1339	−0.5	555[M + K]^+^, 539[M + Na]^+^, **517**[M + H]^+^, 255[M + H-Suc-Glu]^+^	[8,9,20,23,24,26]
	29	Daidzein succinyl glucoside isomer	DSG	19.14	C_25_H_24_O_12_	517.1342	0.3	555[M + K]^+^, 539[M + Na]^+^, **517**[M + H]^+^, 255[M + H-Suc-Glu]^+^	
Phos	1 ^(1)^	Daidzein 7-*O*-phosphate	D7P	8.51	C_15_H_11_O_7_P	335.0315	0.0	**335**[M + H]^+^, 317[M + H − H_2_O]^+^, 255[M + H-Phos]^+^	[14,25]
	2 ^(1)^	Daidzein 4′-*O*-phosphate	D4′P	10.36	C_15_H_11_O_7_P	335.0323	2.4	**335**[M + H]^+^, 317[M + H − H_2_O]^+^, 255[M + H-Phos]^+^	[25]
***Genistein Derivatives (18)***									
Aglycone	38 ^(2)^	Genistein	Gn	23.58	C_15_H_10_O_5_	271.0599	0.7	293[M + Na]^+^, **271**[M + H]^+^	[19,24,26,27,29,33]
Glu	3	Genistein 5-*O*-glucoside	Gn5G	10.76	C_21_H_20_O_10_	433.1131	0.4	471[M + K]^+^, 455[M + Na]^+^, **433**[M + H]^+^, 271[M + H-Glu]^+^	[24,26,27]
	10 ^(2)^	Genistein 7-*O*-glucoside (Genistin)	Gn7G	14.65	C_21_H_20_O_10_	433.1124	−1.2	471[[M + K]^+^, 455[M + Na]^+^, **433**[M + H]^+^, 271[M + H-Glu]^+^	[19,24,26,27,29,33]
Api-Glu	8	Genistein 7-*O*-(6″-*O*-apiosyl)glucoside (Ambocin)	Gn7(6″Ap)G	13.73	C_26_H_28_O_14_	565.1553	0.2	587[M + Na]^+^, **565**[M + H]^+^, 433[M + H-Api]^+^, 271[M + H-Api-Glu]^+^	[24,27]
	9	Genistein 7-*O*-(2″-*O*-apiosyl)glucoside	Gn7(6″Ap)G	14.38	C_26_H_28_O_14_	565.1552	0.0	587[M + Na]^+^, **565**[M + H]^+^, 433[M + H-Api]^+^, 271[M + H-Api-Glu]^+^	[24,27]
Mal-Glu	12	Genistein 5-*O*-(6″-*O*-malonyl)glucoside	Gn5(6″M)G	15.10	C_24_H_22_O_13_	519.1133	0.0	557[M + K]^+^, 541[M + Na]^+^, **519**[M + H]^+^, 271[M + H-Mal-Glu]^+^	[24,26,27]
	25	Genistein 4′-*O*-(6″-*O*-malonyl)glucoside (6″-*O*-Malonylsophoricoside)	Gn4′(6″M)G	18.15	C_24_H_22_O_13_	519.1136	0.5	557[M + K]^+^, 541[M + Na]^+^, **519**[M + H]^+^, 271[M + H-Mal-Glu]^+^	[24,26,27]
	26	Genistein 7-*O*-(4″-*O*-malonyl)glucoside (4″-*O*-Malonylgenistin)	Gn7(4″M)G	18.69	C_24_H_22_O_13_	519.1128	−1.0	557[M + K]^+^, 541[M + Na]^+^, **519**[M + H]^+^, 271[M + H-Mal-Glu]^+^	[24,26,27,30]
	28 ^(2)^	Genistein 7-*O*-(6″-*O*-malonyl)glucoside (6″-*O*-Malonylgenistin)	Gn7(6″M)G	19.14	C_24_H_22_O_13_	519.1130	−0.6	557[M + K]^+^, 541[M + Na]^+^, **519**[M + H]^+^, 271[M + H-Mal-Glu]^+^	[8,24,26,27,29,30,34]
Ac-Glu	22	Genistein 5-*O*-(6″-*O*-acetyl)glucoside	Gn5(6″Ac)G	17.22	C_23_H_22_O_11_	475.1237	−0.4	513[M + K]^+^, 497[M + Na]^+^, **475**[M + H]^+^, 271[M + H-Ac-Glu]^+^	[24]
	30	Genistein 4′-*O*-(6″-*O*-acetyl)glucoside (6″-*O*-Acetylsophoricoside)	Gn4′(6″Ac)G	19.34	C_23_H_22_O_11_	475.1240	0.2	513[M + K]^+^, 497[M + Na]^+^, **475**[M + H]^+^, 271[M + H-Ac-Glu]^+^	[24,26]
	32	Genistein 7-*O*-(4″-*O*-acetyl)glucoside (4″-*O*-Acetylgenistin)	Gn7(4″Ac)G	19.71	C_23_H_22_O_11_	475.1239	0.9	513[M + K]^+^, 497[M + Na]^+^, **475**[M + H]^+^, 271[M + H-Ac-Glu]^+^	[24]
	36 ^(2)^	Genistein 7-*O*-(6″-*O*-acetyl)glucoside (6″-*O*-Acetylgenistin)	Gn7(6″Ac)G	21.79	C_23_H_22_O_11_	475.1238	0.7	513[M + K]^+^, 497[M + Na]^+^, **475**[M + H]^+^, 271[M + H-Ac-Glu]^+^	[24,26,27,29]
Suc-Glu	31	Genistein 7-*O*-(4″-*O*-succinyl)glucoside (4″-*O*-Succinylgenistin)	Gn7(4″S)G	19.62	C_25_H_24_O_13_	533.1289	−0.1	571[M + K]^+^, 555[M + Na]^+^, **533**[M + H]^+^, 271[M + H-Suc-Glu]^+^	[24]
	34	Genistein 7-*O*-(6″-*O*-succinyl)glucoside (6″-*O*-Succinylgenistin)	Gn7(6″S)G	20.31	C_25_H_24_O_13_	533.1288	−0.3	571[M + K]^+^, 555[M + Na]^+^, **533**[M + H]^+^, 271[M + H-Suc-Glu]^+^	[8,9,23,24,26]
	37	Genistein succinyl glucoside isomer	GnSG	22.40	C_25_H_24_O_13_	533.1286	−0.7	571[M + K]^+^, 555[M + Na]^+^, **533**[M + H]^+^, 271[M + H-Suc-Glu]^+^	
Phos	5 ^(1)^	Genistein 7-*O*-phosphate	Gn7P	11.31	C_15_H_11_O_8_P	351.0267	0.8	**351**[M + H]^+^, 333[M + H − H_2_O]^+^, 271[M + H-Phos]^+^	[25]
	7 ^(1)^	Genistein 4′-*O*-phosphate	Gn4′P	12.77	C_15_H_11_O_8_P	351.0268	1.1	**351**[M + H]^+^, 333[M + H − H_2_O]^+^, 271[M + H-Phos]^+^	[25]
***Glycitein Derivatives (6)***									
Aglycone	35 ^(2)^	Glycitein	Gy	21.36	C_16_H_12_O_5_	285.0759	0.5	323[M + K]^+^, 307[M + Na]^+^, **285**[M + H]^+^, 270[M + H-CH_3_]^+^,	[19,24,26,27,29,33]
Glu	6 ^(2)^	Glycitein 7-*O*-glucoside (Glycitin)	Gy7G	12.10	C_22_H_22_O_10_	447.1285	−0.2	485[M + K]^+^, 469[M + Na]^+^, **447**[M + H]^+^, 285[M + H-Glu]^+^	[19,24,26,27,29,33]
Mal-Glu	14	Glycitein 4′-*O*-(6″-*O*-malonyl)glucoside	Gy4′(6″M)G	15.42	C_25_H_24_O_13_	533.1279	−2.0	571[M + K]^+^, 555[M + Na]^+^, **533**[M + H]^+^, 285[M + H-Mal-Glu]^+^	[24,27]
	19 ^(2)^	Glycitein 7-*O*-(6″-*O*-malonyl)glucoside (6″-*O*-Malonylglycitin)	Gy7(6″M)G	16.49	C_25_H_24_O_13_	533.1288	−0.3	571[M + K]^+^, 555[M + Na]^+^, **533**[M + H]^+^, 285[M + H-Mal- Glu]^+^	[8,26,27,29,34]
Ac-Glu	27 ^(2)^	Glycitein 7-*O*-(6″-*O*-acetyl)glucoside (6″-*O*-Acetylglycitin)	Gy7(6″Ac)G	18.69	C_24_H_24_O_11_	489.1394	0.5	527[M + K]^+^, 511[M + Na]^+^, **489**[M + H]^+^, 285[M + H-Ac-Glu]^+^	[24,26,29,36]
Suc-Glu	23	Glycitein 7-*O*-(6″-*O*-succinyl)glucoside (6″-*O*-Succinylglycitin)	Gy7(6″S)G	17.58	C_26_H_26_O_13_	547.1450	0.7	585[M + K]^+^, 569[M + Na]^+^, **547**[M + H]^+^, 285[M + H-Suc-Glu]^+^	[9,24]

All samples analyzed in positive ESI-ionization mode (*m/z* [M + H]^+^) of ToF-MS; [M + Na]^+^ and [M + K]^+^ adduct ions presented. Each peak was tentatively determined by comparing elution order, UV spectra and MS fragmentation provided from previously LC-MS and NMR reports. RT, retention time; Api or Ap (apiose, 132 Da); Glu or G (glucose, 162 Da); Ac (acetyl, 42 Da); Mal or M (malonyl, 86 Da); Suc or S (succinyl, 100 Da); Phos or P (phosphate, 80 Da); D, daidzein (*m/z* 255); Gn, genistein (*m/z* 271); Gy, glycitein (*m/z* 285). ^(1)^ New isoflavone derivatives in this source. ^(2)^ Further confirmed in comparison with authentic standards. ^(3)^ Error (ppm) indicates the mass accuracy of ToF data and its formula expressed as [(calculated ion − observed ion)/calculated ion] × 10^6^ based on *m/z* [M + H]^+^ value.

**Table 2:** Changes in content of thirty-eight isoflavone derivatives during steaming and fermentation (after 21, 36 and 60 h) from the four selected soybean cultivars.

**Acylated Group**	**Peak No.**	**Daewon (Control Variety)**					**KLS 87248 (Korean Landrace)**				
		**Raw**	**Steamed**	**21 h**	**36 h**	**60 h**	**Raw**	**Steamed**	**21 h**	**36 h**	**60 h**
Aglycone	33 ^(2)^	0.2 ± 0.1 ^h^	1.7 ± 0.2 ^ef^	1.6 ± 0.3 ^efg^	10.2 ± 2.5 ^c^	15.8 ± 0.7 ^b^	1.2 ± 0.3 ^efgh^	1.6 ± 0.1 ^efg^	1.7 ± 0.1 ^ef^	15.3 ± 0.3 ^b^	31.7 ± 0.8 ^a^
Glu	4 ^(2)^	7.0 ± 0.6 ^m^	62.2 ± 2.5 ^c^	49.8 ± 0.9 ^e^	25.0 ± 3.8 ^i^	15.3 ± 1.6 ^k^	24.3 ± 1.7 ^j^	107.4 ± 2.2 ^a^	92.9 ± 0.6 ^b^	45.8 ± 0.6 ^f^	23.2 ± 0.6 ^ij^
Mal-Glu	11	4.6 ± 0.2 ^c^	1.8 ± 0.0 ^ij^	1.4 ± 0.1 ^kl^	1.2 ± 0.0 ^lm^	0.9 ± 0.1 ^m^	9.5 ± 0.3 ^a^	4.6 ± 0.5 ^c^	3.7 ± 0.2 ^d^	3.1 ± 0.2 ^fg^	2.3 ± 0.1 ^h^
	13	0.6 ± 0.1 ^g^	0.7 ± 0.1 ^fg^	0.7 ± 0.1 ^fg^	0.9 ± 0.2 ^ef^	1.1 ± 0.2 ^e^	1.5 ± 0.1 ^d^	2.9 ± 0.3 ^a^	2.9 ± 0.2 ^a^	2.9 ± 0.1 ^a^	2.5 ± 0.2 ^b^
	17 ^(2)^	57.5 ± 2.0 ^c^	12.7 ± 0.5 ^h^	13.3 ± 0.6 ^h^	13.3 ± 0.5 ^h^	12.4 ± 1.1 ^h^	116.1 ± 6.1 ^a^	30.8 ± 2.2 ^ef^	30.8 ± 2.1 ^ef^	31.4 ± 1.5 ^ef^	28.4 ± 2.0 ^f^
Ac-Glu	16	ND	0.7 ± 0.0 ^ab^	0.7 ± 0.0 ^abc^	0.8 ± 0.2 ^a^	0.6 ± 0.1 ^cd^	ND	ND	0.5 ± 0.0 ^de^	ND	ND
	24 ^(2)^	ND	5.5 ± 0.3 ^c^	4.0 ± 0.3 ^d^	2.2 ± 0.2 ^f^	1.4 ± 0.1 ^ghi^	0.8 ± 0.1 ^jk^	9.8 ± 0.6 ^a^	8.0 ± 0.6 ^b^	4.4 ± 0.1 ^d^	1.9 ± 0.3 ^fg^
Suc-Glu	15	ND	ND	0.3 ± 0.1 ^e^	0.9 ± 0.1 ^b^	1.0 ± 0.0 ^b^	ND	ND	0.3 ± 0.1 ^de^	1.4 ± 0.1 ^a^	1.4 ± 0.1 ^a^
	18	ND	ND	0.5 ± 0.1 ^h^	1.8 ± 0.5 ^ef^	1.9 ± 0.1 ^de^	ND	ND	1.6 ± 0.2 ^ef^	3.4 ± 0.1 ^b^	4.7 ± 0.3 ^a^
	20	ND	ND	ND	ND	ND	ND	ND	ND	ND	ND
	21	ND	ND	11.1 ± 0.6 ^gh^	14.1 ± 0.5 ^d^	11.7 ± 0.9 ^fg^	ND	ND	13.3 ± 1.0 ^de^	25.7 ± 1.2 ^a^	23.9 ± 1.6 ^b^
	29	3.6 ± 0.4 ^bcd^	0.8 ± 0.5 ^e^	0.8 ± 0.6 ^e^	0.8 ± 0.5 ^e^	0.7 ± 0.5 ^e^	13.8 ± 2.2 ^a^	3.3 ± 0.1 ^bcde^	3.2 ± 0.0 ^bcde^	2.9 ± 0.1 ^cde^	2.6 ± 0.1 ^de^
Phos	1 ^(1)^	ND	ND	1.0 ± 0.0 ^fg^	6.7 ± 0.1 ^de^	19.0 ±1.1 ^b^	ND	ND	1.2 ± 0.2 ^fg^	12.2 ± 0.4	34.6 ± 3.8 ^a^
	2 ^(1)^	ND	ND	ND	ND	ND	ND	ND	ND	0.8 ± 0.0 ^c^	3.6 ± 0.5 ^a^
**Total Daidzein (14)**		**73.6 ± 3.2 ^h^**	**86.1 ± 2.7 ^f^**	**85.1 ± 1.6 ^f^**	**77.9 ± 2.1 ^gh^**	**81.9 ± 3.3 ^fg^**	**167.0 ± 5.5 ^a^**	**160.4 ± 4.4 ^a^**	**160.1 ± 3.8 ^a^**	**149.4 ± 0.9 ^b^**	**160.9 ± 1.7 ^a^**
Aglycone	38 ^(2)^	0.3 ± 0.1 ^efg^	2.0 ± 0.2 ^ef^	1.9 ± 0.0 ^ef^	7.1 ± 2.5 ^c^	15.5 ± 0.7 ^b^	0.7 ± 0.1 ^fg^	1.2 ± 0.1 ^fg^	1.3 ± 0.0 ^efg^	6.9 ± 0.2 ^c^	16.9 ± 0.2 ^a^
Glu	3	0.5 ± 0.0 ^hi^	3.2 ± 0.1 ^c^	3.0 ± 0.1 ^d^	1.6 ± 0.2 ^f^	1.1 ± 0.1 ^g^	1.6 ± 0.4 ^f^	9.0 ± 0.2 ^a^	8.9 ± 0.1 ^a^	4.6 ± 0.1 ^b^	2.5 ± 0.1 ^e^
	10 ^(2)^	9.1 ± 0.7 ^m^	108.0 ± 4.2 ^b^	88.8 ± 2.2 ^d^	57.2 ± 6.2 ^g^	40.6 ± 1.4 ^i^	26.7 ± 0.9 ^k^	122.0 ± 2.4 ^a^	106.0 ± 0.3 ^b^	64.6 ± 0.9 ^f^	39.9 ± 1.1 ^i^
Api-Glu	8	0.2 ± 0.0 ^j^	0.4 ± 0.0 ^hi^	0.4 ± 0.0 ^gh^	0.5 ± 0.0 ^g^	0.5 ± 0.0 ^g^	1.3 ± 0.1 ^a^	0.8 ± 0.0 ^de^	0.8 ± 0.0 ^d^	0.9 ± 0.0 ^c^	1.0 ± 0.1 ^b^
	9	0.4 ± 0.1 ^i^	0.5 ± 0.0 ^hi^	0.6 ± 0.0 ^ghi^	0.6 ± 0.0 ^ghi^	0.6 ± 0.1 ^gh^	1.2 ± 0.0 ^f^	1.3 ± 0.0 ^ef^	1.2 ± 0.0 ^ef^	1.3 ± 0.1 ^ef^	1.4 ± 0.1 ^e^
Mal-Glu	12	3.5 ± 0.2 ^b^	0.8 ± 0.1 ^e^	0.8 ± 0.0 ^e^	0.5 ± 0.1 ^e^	0.5 ± 0.1 ^e^	9.6 ± 0.7 ^a^	1.8 ± 0.3 ^d^	1.8 ± 0.2 ^d^	2.1 ± 0.2 ^cd^	1.8 ± 0.2 ^d^
	25	5.7 ± 0.5 ^d^	1.8 ± 0.4 ^klm^	1.6 ± 0.3 ^mn^	1.1 ± 0.1 ^n^	1.1 ± 0.1 ^n^	10.7 ± 0.8 ^a^	3.7 ± 0.3 ^e^	3.1 ± 0.2 ^efg^	2.5 ± 0.3 ^hij^	1.7 ± 0.2 ^lm^
	26	0.8 ± 0.1 ^e^	0.5 ± 0.0 ^f^	0.5 ± 0.0 ^f^	0.6 ± 0.1 ^f^	0.6 ± 0.1 ^f^	0.3 ± 0.0 ^g^	0.9 ± 0.1 ^e^	0.8 ± 0.1 ^e^	1.0 ± 0.0 ^de^	1.0 ± 0.1 ^de^
	28 ^(2)^	89.3 ± 3.9 ^c^	23.3 ± 1.0 ^g^	24.7 ± 1.5 ^g^	26.1 ± 1.2 ^g^	25.1 ± 2.1 ^g^	123.8 ± 5.1 ^b^	34.8 ± 3.4 ^f^	35.5 ± 3.5 ^f^	38.0 ± 1.7 ^f^	37.3 ± 2.8 ^f^
Ac-Glu	22	ND	1.1 ± 0.1 ^b^	0.7 ± 0.2 ^d^	ND	ND	ND	1.2 ± 0.1 ^a^	1.1 ± 0.0 ^b^	0.8 ± 0.1 ^c^	0.4 ± 0.1 ^e^
	30	ND	0.7 ± 0.1 ^cd^	0.2 ± 0.1 ^e^	ND	ND	ND	1.0 ± 0.2 ^a^	0.7 ± 0.0 ^bc^	0.9 ± 0.2 ^b^	ND
	32	ND	0.9 ± 0.1 ^def^	ND	1.6 ± 0.2 ^b^	1.0 ± 0.1 ^cd^	ND	0.9 ± 0.1 ^def^	1.1 ± 0.1 ^cd^	2.0 ± 0.1 ^a^	1.0 ± 0.0 ^cde^
	36 ^(2)^	0.1 ± 0.0 ^l^	11.1 ± 0.1 ^a^	9.3 ± 0.3 ^c^	6.9 ± 0.5 ^e^	5.2 ± 0.2 ^g^	1.1 ± 0.2 ^k^	11.5 ± 0.2 ^a^	10.1 ± 1.0 ^b^	7.7 ± 0.3 ^d^	4.7 ± 0.6 ^g^
Suc-Glu	31	ND	ND	ND	1.3 ± 0.6 ^de^	3.7 ± 0.3 ^b^	ND	ND	ND	1.3 ± 0.1 ^d^	7.1 ± 0.5 ^a^
	34	ND	ND	17.0 ± 1.0 ^e^	27.0 ± 2.3 ^c^	31.3 ± 2.3 ^b^	ND	ND	14.4 ± 1.5 ^f^	31.0 ± 2.0 ^b^	48.6 ± 3.9 ^a^
	37	1.4 ± 0.1 ^abc^	1.0 ± 0.5 ^c^	0.8 ± 0.5 ^bc^	0.7 ± 0.2 ^bc^	0.9 ± 0.0 ^bc^	2.7 ± 0.0 ^ab^	2.0 ± 0.0 ^abc^	1.4 ± 0.7 ^abc^	1.6 ± 0.0 ^abc^	1.6 ± 0.1 ^abc^
Phos	5 ^(1)^	ND	ND	ND	4.7 ± 0.7 ^cd^	13.2 ± 3.1 ^b^	ND	ND	ND	6.0 ± 0.3 ^c^	14.7 ± 2.5 ^a^
	7 ^(1)^	ND	ND	ND	ND	ND	ND	ND	ND	ND	1.5 ± 0.1
**Total Genistein (18)**		**111.6 ± 5.3 ^f^**	**155.4 ± 4.0 ^d^**	**150.3 ± 2.7 ^d^**	**137.5 ± 3.7 ^e^**	**140.1 ± 7.3 ^e^**	**179.7 ± 3.6 ^bc^**	**192.1 ± 4.7 ^a^**	**188.4 ± 5.4 ^a^**	**173.2 ± 2.8 ^c^**	**183.3 ± 6.6 ^ab^**
Aglycone	35 ^(2)^	ND	0.8 ± 0.1 ^a^	ND	ND	0.6 ± 0.1 ^bcd^	ND	0.9 ± 0.1 ^abc^	0.7 ± 0.0 ^ab^	0.4 ± 0.1 ^cd^	0.7 ± 0.1 ^ab^
Glu	6 ^(2)^	3.5 ± 0.3 ^l^	15.1 ± 0.5 ^c^	14.2 ± 0.1 ^d^	9.3 ± 0.5 ^f^	7.6 ± 0.2 ^h^	8.7 ± 0.5 ^g^	22.3 ± 0.4 ^a^	21.4 ± 0.3 ^b^	14.8 ± 0.1 ^c^	9.2 ± 0.2 ^fg^
Mal-Glu	14	0.9 ± 0.1 ^b^	0.4 ± 0.1 ^fgh^	0.4 ± 0.0 ^fg^	0.3 ± 0.0 ^ghi^	0.3 ± 0.1 ^ghi^	1.2 ± 0.2 ^a^	0.5 ± 0.1 ^efg^	0.5 ± 0.1 ^cde^	0.4 ± 0.1 ^efg^	0.2 ± 0.0 ^i^
	19 ^(2)^	9.5 ± 0.9 ^c^	2.3 ± 0.1 ^g^	2.5 ± 0.1 ^g^	2.6 ± 0.1 ^g^	2.5 ± 0.1 ^g^	17.7 ± 1.4 ^a^	4.9 ± 0.3 ^f^	4.8 ± 0.4 ^f^	5.1 ± 0.2 ^f^	4.8 ± 0.3 ^f^
Ac-Glu	27 ^(2)^	ND	1.2 ± 0.1 ^c^	0.9 ± 0.1 ^d^	0.6 ± 0.1 ^f^	0.3 ± 0.0 ^ijk^	0.1 ± 0.0 ^lm^	1.8 ± 0.2 ^a^	1.4 ± 0.1 ^b^	0.9 ± 0.1 ^d^	0.4 ± 0.0 ^hij^
Suc-Glu	23	ND	ND	1.9 ± 0.1 ^gh^	5.8 ± 0.7 ^d^	7.5 ± 0.5 ^b^	ND	ND	1.5 ± 0.1 ^hi^	7.0 ± 0.2 ^bc^	11.8 ± 1.5 ^a^
**Total Glycitein (6)**		**13.8 ± 1.3 ^g^**	**19.8 ± 0.4 ^d^**	**19.9 ± 0.1 ^d^**	**18.6 ± 0.2 ^de^**	**18.8 ± 1.0 ^de^**	**27.8 ± 1.2 ^bc^**	**30.4 ± 0.4 ^a^**	**30.3 ± 0.9 ^a^**	**28.6 ± 0.5 ^b^**	**27.0 ± 1.9 ^c^**
**Total Isoflavones**		**199.0 ± 9.7 ^h^**	**261.2 ± 6.7 ^f^**	**255.3 ± 4.2 ^f^**	**234.0 ± 5.6 ^g^**	**241.7 ± 11.0 ^g^**	**374.5 ± 7.9 ^b^**	**382.9 ± 9.5 ^a^**	**378.8 ± 10.1 ^ab^**	**351.3 ± 4.2 ^c^**	**371.2 ± 9.1 ^ab^**
**Acylated Group**	**Peak No.**	**Nongrim 51 (Japanese Breeding Line)**					**GNU-2007-14613 (Korean Landrace)**				
		**Raw**	**Steamed**	**21 h**	**36 h**	**60 h**	**Raw**	**Steamed**	**21 h**	**36 h**	**60 h**
Aglycone	33 ^(2)^	0.2 ± 0.1 ^gh^	1.8 ± 0.0 ^ef^	1.4 ± 0.2 ^efgh^	6.0 ± 0.3 ^d^	16.1 ± 1.0 ^b^	1.0 ± 0.0 ^fgh^	1.3 ± 0.1 ^efgh^	0.9 ± 0.1 ^fgh^	2.5 ± 0.3 ^e^	9.9 ± 0.1 ^c^
Glu	4 ^(2)^	13.1 ± 0.5 ^kl^	64.3 ± 1.1 ^c^	55.0 ± 0.3 ^d^	36.5 ± 0.8 ^g^	24.2 ± 1.0 ^ij^	10.1 ± 2.1 ^l^	30.8 ± 1.4 ^h^	25.1 ± 0.6 ^i^	15.7 ± 0.7 ^k^	12.0 ± 0.3 ^l^
Mal-Glu	11	5.5 ± 0.3 ^b^	4.6 ± 0.2 ^c^	3.5 ± 0.2 ^de^	3.3 ± 0.2 ^ef^	2.8 ± 0.2 ^g^	3.8 ± 0.1 ^d^	2.8 ± 0.2 ^g^	2.3 ± 0.1 ^h^	2.1 ± 0.1 ^hi^	1.6 ± 0.2 ^jk^
	13	0.7 ± 0.0 ^fg^	2.0 ± 0.1 ^c^	2.0 ± 0.4 ^c^	2.4 ± 0.2 ^b^	2.6 ± 0.4 ^ab^	0.5 ± 0.1 ^g^	1.7 ± 0.1 ^d^	1.6 ± 0.1 ^d^	1.6 ± 0.2 ^d^	1.7 ± 0.1 ^cd^
	17 ^(2)^	92.0 ± 2.0 ^b^	31.1 ± 1.5 ^ef^	30.1 ± 1.8 ^ef^	33.0 ± 2.3 ^e^	29.9 ± 0.8 ^ef^	48.3 ± 2.1 ^d^	21.7 ± 1.5 ^g^	22.2 ± 1.1 ^g^	22.9 ± 1.1 ^g^	19.7 ± 1.6 ^g^
Ac-Glu	16	ND	0.4 ± 0.0 ^ef^	ND	ND	ND	ND	0.3 ± 0.1 ^fg^	0.3 ± 0.0 ^g^	0.6 ± 0.0 ^bcd^	ND
	24 ^(2)^	ND	3.3 ± 0.2 ^e^	2.8 ± 0.3 ^e^	2.2 ± 0.2 ^f^	1.4 ± 0.2 ^ghi^	0.3 ± 0.0 ^kl^	1.5 ± 0.2 ^gh^	1.3 ± 0.2 ^hi^	1.0 ± 0.1 ^ij^	0.6 ± 0.1 ^jk^
Suc-Glu	15	ND	ND	0.4 ± 0.2 ^d^	0.9 ± 0.0 ^b^	0.9 ± 0.1 ^b^	ND	ND	ND	ND	0.7 ± 0.1 ^c^
	18	ND	1.0 ± 0.1 ^g^	1.5 ± 0.3 ^f^	2.3 ± 0.2 ^d^	2.6 ± 0.3 ^c^	ND	ND	1.1 ± 0.2 ^g^	1.7 ± 0.1 ^ef^	2.3 ± 0.2 ^d^
	20	ND	ND	ND	0.2 ± 0.0 ^b^	0.5 ± 0.0 ^a^	ND	ND	ND	ND	ND
	21	ND	ND	9.3 ± 0.6 ^i^	15.8 ± 1.0 ^c^	12.8 ± 0.7 ^ef^	ND	ND	5.8 ± 0.3 ^j^	10.0 ± 0.5 ^hi^	10.1 ± 1.0 ^hi^
	29	5.4 ± 0.1 ^bc^	3.7 ± 0.2 ^bcde^	3.6 ± 0.1 ^bcde^	3.4 ± 0.1 ^bcde^	3.0 ± 0.1 ^bcde^	5.9 ± 0.2 ^b^	2.6 ± 0.1 ^de^	2.1 ± 0.9 ^de^	2.4 ± 0.2 ^de^	1.5 ± 0.6 ^de^
Phos	1 ^(1)^	ND	ND	0.5 ± 0.1 ^fg^	7.8 ± 0.3 ^d^	19.0 ± 1.1 ^b^	ND	ND	ND	1.9 ± 0.3 ^f^	6.0 ± 0.2 ^e^
	2 ^(1)^	ND	ND	ND	ND	1.3 ± 0.1 ^b^	ND	ND	ND	ND	ND
**Total Daidzein (14)**		**116.9 ± 2.8 ^cd^**	**112.2 ± 0.5 ^de^**	**110.2 ± 1.7 ^e^**	**113.7 ± 3.0 ^cde^**	**117.1 ± 3.4 ^c^**	**69.8 ± 2.4 ^i^**	**62.6 ± 2.3 ^j^**	**62.6 ± 0.5 ^j^**	**62.5 ± 1.1 ^j^**	**66.1 ± 2.2 ^ij^**
Aglycone	38 ^(2)^	0.2 ± 0.0 ^fg^	2.2 ± 0.2 ^e^	2.2 ± 0.3 ^e^	4.4 ± 0.2 ^d^	14.8 ± 0.2 ^b^	0.9 ± 0.1 ^efg^	1.4 ± 0.1 ^efg^	1.4 ± 0.1 ^efg^	2.0 ± 0.1 ^ef^	6.3 ± 0.1 ^c^
Glu	3	0.3 ± 0.0 ^i^	1.0 ± 0.1 ^g^	1.0 ± 0.1 ^g^	0.6 ± 0.0 ^h^	0.4 ± 0.1 ^hi^	0.4 ± 0.1 ^hi^	1.4 ± 0.0 ^f^	1.4 ± 0.1 ^f^	0.9 ± 0.0 ^g^	0.6 ± 0.1 ^h^
	10 ^(2)^	18.7 ± 0.9 ^l^	104.4 ± 2.1 ^b^	95.1 ± 1.4 ^c^	72.1 ± 1.5 ^e^	53.7 ± 2.3 ^gh^	15.0 ± 3.4 ^l^	49.8 ± 2.7 ^h^	41.7 ± 1.3 ^i^	31.8 ± 1.5 ^j^	28.2 ± 0.4 ^j^
Api-Glu	8	0.5 ± 0.0 ^g^	0.5 ± 0.0 ^g^	0.7 ± 0.0 ^f^	0.7 ± 0.0 ^ef^	0.8 ± 0.0 ^de^	0.3 ± 0.1 ^ij^	ND	ND	ND	ND
	9	5.5 ± 0.1 ^d^	5.5 ± 0.1 ^d^	5.7 ± 0.1 ^c^	5.9 ± 0.3 ^b^	6.2 ± 0.3 ^a^	0.6 ± 0.0 ^gh^	0.6 ± 0.0 ^gh^	0.6 ± 0.0 ^gh^	0.7 ± 0.0 ^gh^	0.8 ± 0.0 ^g^
Mal-Glu	12	2.0 ± 0.1 ^cd^	0.9 ± 0.1 ^e^	0.9 ± 0.0 ^e^	0.8 ± 0.1 ^e^	0.6 ± 0.1 ^e^	2.3 ± 0.1 ^c^	0.8 ± 0.1 ^e^	0.8 ± 0.1 ^e^	0.9 ± 0.1 ^e^	0.8 ± 0.1 ^e^
	25	8.5 ± 0.2 ^b^	3.5 ± 0.4 ^ef^	3.0 ± 0.3 ^fgh^	3.1 ± 0.4 ^fgh^	2.9 ± 0.2 ^ghi^	6.3 ± 0.4 ^c^	2.7 ± 0.2 ^ghij^	2.4 ± 0.2 ^ijk^	2.2 ± 0.2 ^jkl^	1.8 ± 0.2 ^klm^
	26	1.6 ± 0.1 ^c^	1.8 ± 0.1 ^b^	1.6 ± 0.3 ^bc^	2.0 ± 0.2 ^a^	2.2 ± 0.1 ^a^	0.1 ± 0.0 ^g^	1.0 ± 0.1 ^de^	1.0 ± 0.1 ^de^	1.0 ± 0.1 ^de^	1.1 ± 0.2 ^d^
	28 ^(2)^	135.5 ± 8.1 ^a^	53.5 ± 3.8 ^e^	53.0 ± 4.1 ^e^	59.2 ± 5.0 ^e^	55.9 ± 4.0 ^e^	81.1 ± 5.4 ^d^	40.2 ± 1.5 ^f^	39.3 ± 2.9 ^f^	41.2 ± 2.7 ^f^	35.4 ± 0.4 ^f^
Ac-Glu	22	ND	ND	ND	ND	ND	ND	ND	ND	ND	ND
	30	ND	0.8 ± 0.1 ^b^	0.6 ± 0.1 ^cd^	0.5 ± 0.1 ^d^	ND	ND	ND	ND	ND	ND
	32	ND	0.5 ± 0.0 ^g^	0.6 ± 0.0 ^f^	1.2 ± 0.3 ^c^	0.8 ± 0.1 ^ef^	ND	0.3 ± 0.1 ^h^	ND	0.5 ± 0.1 ^g^	0.3 ± 0.0 ^gh^
	36 ^(2)^	0.2 ± 0.0 ^l^	7.3 ± 0.1 ^de^	7.0 ± 0.2 ^e^	6.2 ± 0.3 ^f^	4.7 ± 0.2 ^g^	1.0 ± 0.0 ^kl^	3.5 ± 0.1 ^h^	3.2 ± 0.2 ^hi^	2.8 ± 0.0 ^i^	2.0 ± 0.0 ^j^
Suc-Glu	31	ND	ND	ND	0.9 ± 0.2 ^ef^	3.4 ± 0.4 ^bc^	ND	ND	ND	0.6 ± 0.2 ^f^	3.3 ± 0.4 ^c^
	34	ND	ND	13.4 ± 0.8 ^f^	26.0 ± 2.1 ^c^	30.7 ± 1.4 ^b^	ND	ND	7.2 ± 0.4 ^g^	13.8 ± 1.4 ^f^	22.4 ± 2.0 ^d^
	37	1.1 ± 0.4 ^abc^	3.0 ± 0.1 ^a^	2.9 ± 0.2 ^a^	2.5 ± 0.1 ^ab^	2.4 ± 0.2 ^abc^	1.6 ± 0.1 ^abc^	2.2 ± 0.1 ^abc^	2.0 ± 0.1 ^abc^	1.8 ± 0.1 ^abc^	1.5 ± 0.0 ^abc^
Phos	5 ^(1)^	ND	ND	ND	4.2 ± 0.2 ^d^	12.0 ± 0.6 ^b^	ND	ND	ND	0.9 ± 0.1 ^f^	2.6 ± 0.2 ^e^
	7 ^(1)^	ND	ND	ND	ND	ND	ND	ND	ND	ND	ND
**Total Genistein (18)**		**174.1 ± 8.0 ^c^**	**184.8 ± 1.7 ^a^**	**187.7 ± 4.0 ^a^**	**190.4 ± 7.7 ^a^**	**191.6 ± 3.7 ^a^**	**109.6 ± 1.2 ^fg^**	**103.9 ± 2.2 ^g^**	**101.0 ± 2.0 ^g^**	**101.2 ± 3.1 ^g^**	**107.2 ± 5.3 ^fg^**
Aglycone	35 ^(2)^	ND	ND	ND	ND	ND	ND	ND	ND	ND	0.4 ± 0.0 ^d^
Glu	6 ^(2)^	2.0 ± 0.2 ^m^	5.9 ± 0.1 ^i^	6.1 ± 0.0 ^i^	4.4 ± 0.1 ^k^	3.80 ± 0.1 ^l^	4.7 ± 0.6 ^jk^	10.0 ± 0.5 ^e^	8.8 ± 0.2 ^fg^	6.1 ± 0.2 ^i^	5.1 ± 0.1 ^j^
Mal-Glu	14	0.6 ± 0.1 ^c^	0.2 ± 0.0 ^hi^	ND	ND	ND	0.9 ± 0.1 ^b^	0.6 ± 0.0 ^cd^	0.5 ± 0.0 ^cdef^	0.6 ± 0.1 ^cde^	0.5 ± 0.1 ^defg^
	19 ^(2)^	5.3 ± 0.5 ^ef^	2.2 ± 0.1 ^g^	2.3 ± 0.1 ^g^	2.3 ± 0.1 ^g^	2.5 ± 0.0 ^g^	11.5 ± 0.6 ^b^	6.0 ± 0.1 ^de^	5.9 ± 0.4 ^de^	6.3 ± 0.3 ^d^	5.2 ± 0.3 ^f^
Ac-Glu	27 ^(2)^	ND	0.6 ± 0.0 ^fg^	0.4 ± 0.0 ^ghi^	0.3 ± 0.0 ^jk^	0.2 ± 0.0 ^lm^	0.1 ± 0.0 ^mn^	0.9 ± 0.0 ^d^	0.7 ± 0.0 ^e^	0.5 ± 0.1 ^fgh^	0.2 ± 0.0 ^kl^
Suc-Glu	23	ND	ND	ND	2.2 ± 0.2 ^g^	3.1 ± 0.2 ^f^	ND	ND	1.1 ± 0.1 ^i^	4.0 ± 0.2 ^e^	6.7 ± 0.6 ^c^
**Total Glycitein (6)**		**8.0 ± 0.7 ^i^**	**8.9 ± 0.1 ^hi^**	**8.8 ± 0.2 ^hi^**	**9.2 ± 0.4 ^hi^**	**9.7 ± 0.2 ^h^**	**17.1 ± 0.7 ^f^**	**17.5 ± 0.4 ^ef^**	**17.0 ± 0.4 ^f^**	**17.5 ± 0.5 ^ef^**	**18.0 ± 1.0 ^ef^**
**Total Isoflavones**		**299.0 ± 10.4 ^e^**	**305.9 ± 2.0 ^de^**	**306.6 ± 5.8 ^de^**	**313.3 ± 11.0 ^d^**	**318.3 ± 6.1 ^d^**	**196.5 ± 2.5 ^hi^**	**184.0 ± 4.8 ^ij^**	**180.6 ± 2.6 ^j^**	**181.2 ± 4.3 ^j^**	**191.3 ± 8.6 ^hij^**

Each value expressed as mean ± SD (*n* = 3). The quantification (mg/100 g, dry weight) for each peak carried out using pre-inserted internal standard (6-methoxyflavone). Different small letters in the same low with mean values indicate a significant difference at *p* < 0.05 by Duncan’s multiple range test. ND, not detected. ^(^^1)^ New isoflavone derivatives in this source. ^(^^2)^ Further confirmed in comparison with authentic standards. Bold numbers: importance and recognition of total contents.

## Data Availability

Not applicable.

## Sample Availability

Samples of the compounds are not available from the authors.
